# Whole genome characterization of reassortant G10P[11] strain (N155) from a neonate with symptomatic rotavirus infection: Identification of genes of human and animal rotavirus origin

**DOI:** 10.1016/j.jcv.2009.05.003

**Published:** 2009-07

**Authors:** Sasirekha Ramani, Miren Iturriza-Gomara, Atanu Kumar Jana, Kurien Anil Kuruvilla, James J. Gray, David W. Brown, Gagandeep Kang

**Affiliations:** a Department of Gastrointestinal Sciences, Christian Medical College, Vellore 632004, India; b Virus Reference Department, Centre for Infection, Health Protection Agency, London, United Kingdom; c Department of Neonatology, Christian Medical College, Vellore, India

**Keywords:** Rotavirus, Neonates, Gastrointestinal disease, Whole genome characterization

## Abstract

**Background:**

Rotavirus G10P[11] strains have long been associated with asymptomatic neonatal infections in some parts of India. We have previously reported G10P[11] strains associated with both asymptomatic infections and severe gastrointestinal disease in neonates from Vellore in southern India, with >90% partial nucleotide and amino acid identity to the VP4, VP6, VP7 and NSP4 genes of the exclusively asymptomatic G10P[11] strain I321.

**Objectives:**

In this study, the whole genome of a G10P[11] isolate (N155) from a neonate with severe gastrointestinal disease was characterized to determine whether there were significant differences in its genetic makeup in comparison to G10P[11] strain I321 and to establish the origin of the G10P[11] strains in Vellore.

**Study design:**

PCR amplification and complete genome sequencing was carried out for all 11 gene segments of symptomatic G10P[11] rotavirus isolate N155. Nucleotide and amino acid sequence similarity with I321, other human and bovine strains for each gene segment were determined. The origin of each gene was determined based on the degree of identity to bovine or human rotavirus strains.

**Results:**

N155 was found to be a reassortant between human and bovine rotaviruses. With the exception of NSP2, gene sequences of strain N155 showed >90% identity to published sequences of I321. Gene segments encoding NSP1, 2 and 3 were of human rotavirus origin for both strains; however, phylogenetic analysis of NSP2 sequences indicated that the human parental strain that led to the origin of these bovine-human reassortant strains was different. There were no significant differences between NSP2 sequences of strains from symptomatic and asymptomatic neonates in the same setting.

**Conclusions:**

The study shows that the difference in clinical presentations in neonates may not be due to the limited variability in the genome sequence of G10P[11] strains and that G10P[11] strains in different parts of India could have evolved through reassortment of different parental strains.

## 1 Background

Rotaviruses have an 11 segmented double-stranded RNA genome encoding 6 structural and 6 non-structural proteins.^1^ Classification into 19 G and 27 P genotypes is based on variability in genes encoding outer capsid proteins VP7 and VP4.^1–6^

Bovine–human reassortant G10P[11] rotaviruses were first described in asymptomatic neonatal infections in Bangalore and Mysore in southern India.^7^ A significant reduction in rotavirus diarrhea hospitalizations was reported in parallel with high rates of asymptomatic neonatal rotavirus infection.^8^ The strain I321, with all genes of bovine rotavirus origin except NSP1 and NSP3^9^ was developed as a candidate vaccine, but later withdrawn because of poor immunogenicity.^10^ The other Indian candidate vaccine 116E is also a natural bovine–human reassortant (human G9 VP7 and bovine P[11] VP4) isolated from neonates.^11,12^

I321-like G10P[11] strains causing both symptomatic and asymptomatic infections have been described in neonates from Vellore, southern India.^13,14^ These strains were seen in neonates with diarrhea, vomiting and other gastrointestinal symptoms. Partial genome analysis of NSP4, VP6, VP7 and VP4 did not reveal significant differences between G10P[11] strains from symptomatic and asymptomatic infections or from strain I321.^13^

## 2 Objectives

All genes from N155, a G10P[11] rotavirus isolate from a symptomatic child, were compared with published data on I321, a culture-adapted asymptomatic strain developed as a vaccine, to study potential determinants of virulence or attenuation.

## 3 Study design and methods

### 3.1 Preliminary studies

Stool samples from 245 neonates in the nurseries of the Christian Medical College were screened for rotavirus using an enzyme immunoassay (EIA) for VP6 antigen (Rota IDEIA, Oxoid, Basingstoke, UK).^14^ Viral RNA was extracted from 20% fecal suspensions in minimum essential medium (MEM) using the guanidine isothiocyanate-silica based method.^15^ Complementary DNA (cDNA) was generated using random primers (hexamers; Pd(N)6, Pharmacia Biotech, UK) and reverse transcription with 400 units of Moloney murine leukemia virus reverse transcriptase (M-MLV RT) (Invitrogen, UK). The sample were genotyped by RT-PCR for VP7 and VP4 genes as previously described.^16–20^ Briefly, for G-typing, the first round PCR used primers VP7-F and VP7-R primers to amplify an 881 bp region of the VP7 gene. The nested multiplex PCR incorporated primers specific for amplification of genotypes G1, G2, G3, G4, G8, G9 and G10. Primers Con2 and Con3 were used in the first round PCR to amplify an 876 bp fragment of the VP4 gene. The second round PCR included primers specific for genotypes P[4], P[6], P[8], P[9], P[10] and P[11]. Ninety samples were positive for rotavirus and >80% of strains were genotyped G10P[11]. Polyacrylamide gel electrophoresis of a subset of G10P[11] strains from symptomatic and asymptomatic neonates did not show any differences in RNA migration pattern.

### 3.2 Source of rotavirus strain for complete genome characterization

N155 RNA was obtained from the stool sample of a term, male baby born by normal vaginal delivery at 34 + 1 weeks of gestation, who was referred to the nursery at 67 h of age with abdominal distension and feed intolerance. An abdominal radiograph revealed dilated bowel loops and NEC. The baby was given intravenous fluids and kept nil oral for three days after which time feeds were initiated and well tolerated. The sample was screened for other enteropathogens, including Norovirus genogroups I and II, enteric adenovirus, *Cryptosporidium* spp. and diarrhoeagenic *E. coli*, including enteropathogenic, enterohaemorrhagic, enteroinvasive, diffusely adherent, enteroaggregative and enterotoxigenic *E. coli* using published oligonucleotide primers and methods.^21^

### 3.3 Genome characterization

RNA extraction and random priming reverse transcription for cDNA synthesis was carried out as described previously. Primers for the amplification of individual genes were designed using primer3 (http://frodo.wi.mit.edu/) and BioEdit softwares (http://www.mbio.ncsu.edu/BioEdit/bioedit.html) based on corresponding genes sequences available in GenBank. Primer pairs were designed to amplify short fragments of each gene ranging from 387 to 1355 bp, with each fragment overlapping the next one by about 100 bp. RT-PCR was carried out with an initial denaturation at 95 °C for 5 min and 35 cycles of amplification using 5U of Taq polymerase (Invitrogen). High-fidelity Taq polymerase (Invitrogen) was used for the amplification of three genes (VP6, VP7 and NSP3) whose amplicon sizes were >1000 bp. The final sequence was determined by assembling consensus genome sequences from all fragments for the gene. The primer sequences used for sequencing the beginning and end of each gene in this study are given in Table 1. All primers used in the study and their sequences are listed in the supplementary table.

### 3.4 Determination of 5′ and 3′ terminal sequences

The 5′ and 3′ sequences of the 11 gene segments were determined by using the single-primer amplification method.^22^ Extraction of viral RNA was followed by ligation of a modified amino-linked oligonucleotide (5′-PO_4_-TTCCTTATGCAGCTGATCACTCTGTGTCA-C_6_-NH_2_-3′) to the 3′ ends of both strands of the viral dsRNA using T4 RNA Ligase (Promega, USA). One step RT-PCR using Superscript III RT-PCR kit (Invitrogen, UK) was carried out with a primer complementary to the modified amino-linked oligonucleotide (5′-TGACACAGAGTGATCAGC-3′) and a gene specific internal primer.

### 3.5 Nucleotide sequencing and analysis

PCR amplicons were purified using PCR cleanup filter plates (Millipore, USA) and sequenced with ABI PRISM Big Dye Terminator Cycle Sequencing Ready Reaction kit (Applied Biosystems, USA). Sequences were resolved in the ABI PRISM 310 Genetic Analyzer (Applied Biosystems, USA). Deduced amino acid (aa) sequences were derived using the Expert Protein Analysis System (ExPASy) proteomics server of the Swiss Institute of Bioinformatics (SIB). The nucleotide (nt) and deduced amino acid sequences of all genes were compared with sequences available in the NCBI (National Center for Biotechnology Information) GenBank database using BLAST (Basic Local Alignment Search Tool). Multiple alignments and phylogenetic analysis of nt and deduced aa sequences were performed using Clustal W and neighbor-joining algorithms of the BioEdit (version 7.0.5.3) or Bionumerics (Applied Maths, Kortrijk, Belgium) software packages. Dendrograms were confirmed by neighbor-joining and maximum parsimony methods. Genetic lineages were confirmed by bootstrap values of >95% using 1000 pseudoreplicate runs.

### 3.6 Sequence accession numbers

The nucleotide sequences of the 11 gene segments of N155 have been deposited in GenBank under the accession numbers EU200793 to EU200803.

## 4 Results

### 4.1 Nucleotide sequencing and comparison with I321

A total of 18,242 nucleotides encoding 5781 amino acids of the eleven segments of the G10P[11] strain N155 were sequenced. The complete coding sequences (cds) were determined for all genes except VP6 and NSP3, in which the first two and three amino acids, respectively, of the open reading frames could not be ascertained.

N155 sequences were compared with published sequences of corresponding genes, with origin determined based on the degree of identity to bovine or human rotavirus strains. The closest sequence match and similarity with I321, human and bovine strains for each gene of N155 is shown in Table 2. Sequence data of I321 was available only for genes VP4, VP6, VP7, NSP1, NSP2, NSP3 and NSP4. Comparison of nt sequences of N155 showed >93% identity for all genes except NSP2.

### 4.2 Structural proteins

The VP1, VP2 and VP3 genes of N155 were of bovine origin, with 93%, 94% and 92% identity, respectively, with bovine strains NCDV or UK. The VP6 sequence of strain N155 clustered with SGI rotavirus strains and was more closely related to I321 (97% identity at the nucleotide (nt) level) and VP6 SGI sequences derived from bovine strains than SGI sequences of common human rotavirus strains (Table 2). On translation, there were 4 amino acid (aa) substitutions between I321 and N155 at positions 58, 179, 190 and 244, none of which are believed to be subgroup-defining (data not shown).^23^ The genes encoding VP4 and VP7 were more closely related to strain I321 (95% and 93% at the nt level, respectively) than to other bovine or human P[11] and G10 strains (Table 2).

### 4.3 Nonstructural proteins

**Fig. 1:**
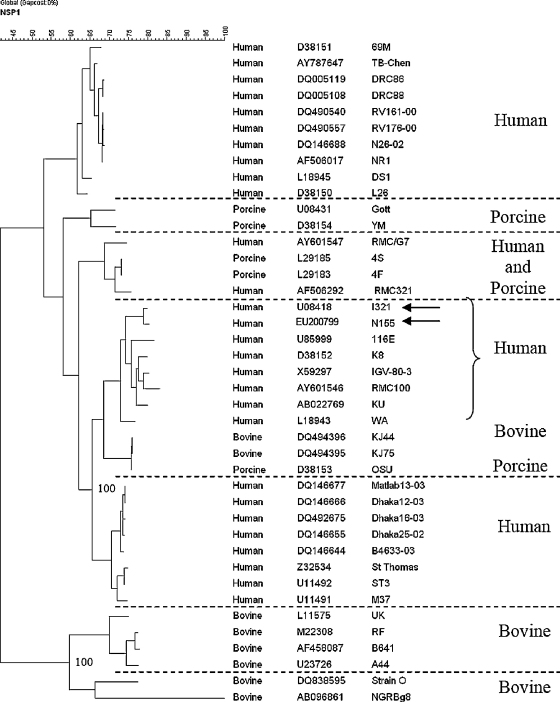
Phylogenetic dendrogram constructed by maximum parsimony method based on nucleotide sequences of gene encoding NSP1 of bovine, porcine and human rotavirus strains. Bootstrap values for 1000 pseudoreplicates are shown. Strains N155 and I321 are identified by arrows. The host species of origin for distinct genetic lineages are indicated. The origin of the strain, accession number and strain name are given in columns 1–3. N155 and I321 are in the same lineage. There are 2 clusters within this lineage, one with bovine and porcine rotavirus strains more closely related to human strains than to other animal strains in separate lineages.

The gene encoding NSP1 of N155 was 97% identical to the corresponding sequence of I321, and likely of human origin (Table 2). Human rotavirus strains clustered in four genetic lineages with strains N155 and I321 in the same lineage (Fig. 1). The derived aa sequences showed 15 substitutions between the two strains, one of which was located in the cysteine rich zinc finger motif (I321, R55K N155). Mutations in cysteine and histidine residues in the zinc finger motif are important in NSP1 stability and interferon regulatory factor 3 (IRF3) binding,^24^ and multipoint substitutions of arginine–lysine residues increase enzyme thermostability.^25,26^ However, a single substitution of these highly similar basic amino acids is unlikely to result in a significant difference in the strain. This is supported by the finding that this substitution was conserved among symptomatic and asymptomatic G10P[11] strains from Vellore (data not shown).

**Fig. 2:**
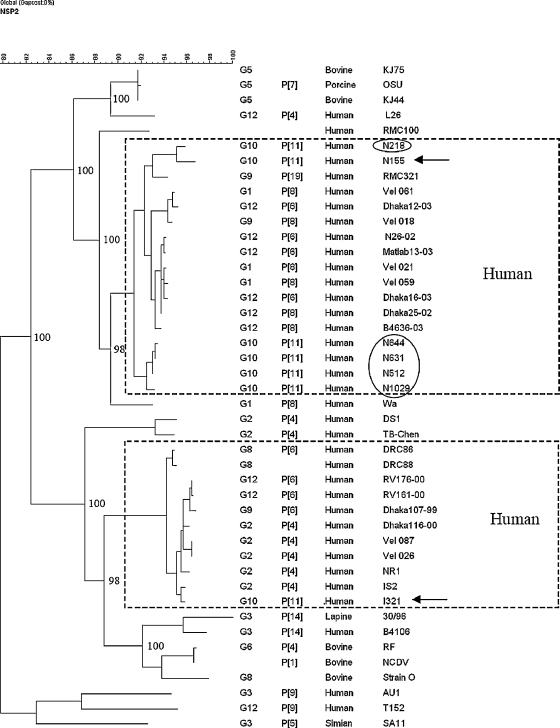
Phylogenetic dendrogram constructed by maximum parsimony method based on nucleotide sequences of gene encoding NSP2. Bootstrap values for 1000 pseudo replicates are shown. Most human rotavirus strains clustered within two distinct genetic lineages that are indicated by boxes. Strains N155 and I321 belonged to different lineages and are identified by arrows. The VP7 and VP4 genotype, origin of the strain and strain name are given in columns 1–4. The circle indicates additional G10P[11] strains from Vellore (N218, N512, N631, N644 and N1029).

The NSP2 encoding gene of N155 was closely related to sequences from human rotaviruses showing >95% nt and 98% aa identity with recent human isolates from Vellore and Bangladesh of common genotypes (G1P[8] and G9P[8]), and >92% with human prototype strain Wa. Only 80.9% nt identity was seen with I321 and 82.8–89.1% with bovine strains (Table 2). Phylogenetic analysis of the NSP2 sequences showed that most human rotaviruses segregated into 2 genetic clusters and N155 and I321 clustered in different lineages of human rotaviruses (Fig. 2).

**Fig. 3:**
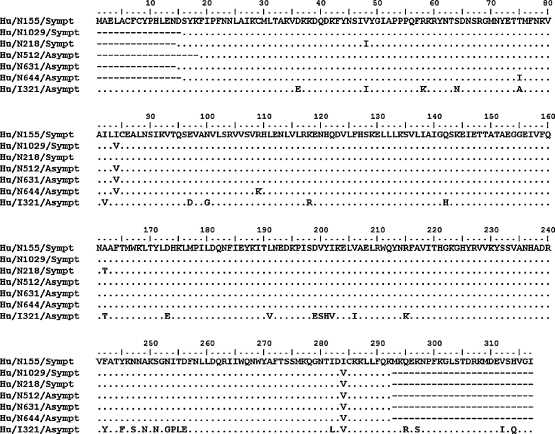
Alignment of deduced aa sequences of NSP2 gene of G10P[11] strains from asymptomatic and symptomatic neonates from Vellore. The aa sequence of strain N155 is shown on top. The aa sequence of strain I321 has also been included for comparison. The residues at which amino acid substitutions occur is indicated for other G10P[11] isolates.

In order to assess the role of this difference in virulence, partial nucleotide sequence analysis of the region encoding aa 15–291 was carried out from 5 additional G10P[11] strains (2 symptomatic and 3 from asymptomatic infections). No significant differences were seen among NSP2 sequences of these isolates (Fig. 3). Terminal sequencing reactions were not carried out for the additional G10P[11] sequences as these regions are known to be highly conserved.

**Fig. 4:**
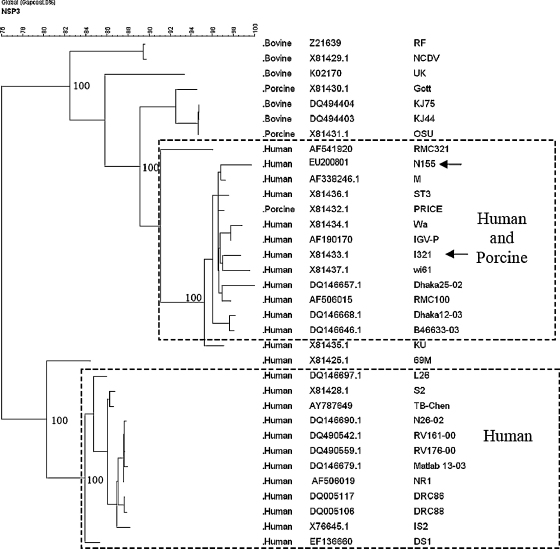
Phylogenetic dendrogram constructed by maximum parsimony method based on nucleotide sequences of gene encoding NSP3. Bootstrap values for 1000 pseudo replicates are shown. Strains N155 and I321 are identified by arrows. Two distinct genetic lineages of human rotavirus strains were identified, with one lineage more closely related to animal rotavirus strains. Both I321 and N155 belong to this lineage. The origin of the strain, accession number and strain name are given in columns 1–3.

The NSP3 gene of N155 was similar to I321 (>95% identity at the nt level) and was highly related to human rotaviruses (Table 2). Two distinct genetic lineages were observed among human rotavirus NSP3 gene sequences (Fig. 4).

The NSP4 sequence of N155 showed 95% identity with I321 and other G10P[11] strains isolated in Vellore (Table 2), and were likely to originate from a bovine strain.^13^ One aa substitution (I321 C129R N155) in the enterotoxin coding region and substitutions at aa residues 97, 94 and 136 described as associated with virulence among lapine strain BAP, porcine strains Gottfried and OSU, respectively^27,28^ were seen; however, these changes in N155 were conserved across isolates from both symptomatic and asymptomatic G10P[11] strains from Vellore.^13^

Gene segment 11 of strain N155 showed 95% identity at the nt level to bovine strain VMRI (Table 2).

## 5 Discussion

Sequence analysis of the complete genome of strain N155 isolated from a neonate with symptomatic rotavirus infection confirms that G10P[11] strains found in humans in Vellore are bovine–human reassortant strains. Eight out of 11 gene segments are likely to have originated from bovine strains. With the exception of NSP2, a high degree of identity was seen between N155 and I321 in six of seven genes for which sequence data was available for both strains. Sequence data on I321 for genes encoding VP1, VP2, VP3 and NSP5 were not available for comparison although these have been reported to be of bovine origin through whole genome hybridization studies.^9^

This study showed that the NSP2 gene of both I321 and N155 originated from human rotavirus strains, but the strains belonged to two distinct genetic lineages with <80% identity between them. These findings would suggest that independent reassortment events between human and bovine strains led to the emergence of G10P[11] strains in Vellore and Bangalore. Both strains have three genes of human rotavirus origin, but the gene encoding NSP2 clearly originated from different human parental strains. These differences however do not explain the differences in virulence, as shown through more extensive analysis of NSP2 sequences from additional G10P[11] strains from symptomatic and asymptomatic infections.

Early studies on I321 had revealed strong cross hybridization of NSP1, NSP2 and NSP3 genes with human rotavirus strain Wa.^9^ Subsequent sequence analysis showed that NSP1 and NSP3 genes were of human origin,^29,30^ and NSP2 was assigned to a bovine origin. The current study has demonstrated that the NSP2 of strains I321 clusters within a different genetic lineage of human rotaviruses. The whole genome hybridization methods for tracking the origin of rotavirus gene segments may be insensitive, probably due to difficulties in standardizing appropriately stringent conditions.

Based on the whole genome characterization of G10P[11], the 3 genes of human rotavirus origin—NSP1, NSP2 and NSP3 are probably essential for efficient replication of a bovine strain in a human host. The role of these genes in host restriction has previously been discussed in other studies but the data are inconclusive.^29,31–33^ In this study, segregation of NSP1 and NSP3 into genetic lineages did not correlate with the host of origin but there was more clear segregation into genetic lineages and clusters according to the host of origin for NSP2.

Complete genome sequences are available from few strains throughout the world^34–36^ and there is lack of information on co-circulating animal rotaviruses. The comparison of N155 with I321 is important because G10P[11] strains in Vellore are associated with disease,^14^ while I321 was developed as a vaccine candidate because of reports of exclusive asymptomatic infection.^7^ Further studies involving complete genome characterization and amino acid modifications for each gene and their role in structure and function from a number of symptomatic and asymptomatic G10P[11] strains will help with assessment of virulence determinants. Co-surveillance of animal and human strains and more comprehensive sequence analyses would provide a better understanding of the emergence and evolution of rotavirus strains. Host factors such as difference in susceptibility and response to infection, maternal protection and innate immune response may also play a role in determining disease.

## Conflict of interest

None.

## Figures and Tables

**Table 1:** Forward and reverse primers used for the amplification of the 11 gene segments.

Gene	Primer	Sequences
VP1	Forward	CTCACAATCCGCAGTTCAAA
	Reverse	TCGCATTGGTATACGGTGAA
VP2	Forward	AAGGTTCAATGGCGTACAGG
	Reverse	CGATACGAATGCAAGCAGAT
VP3	Forward	GGCTCAGGTATATGCGGACA
	Reverse	TCACGATGTGACCAGTGTGTT
VP4	Forward	TGGCTTCGCCATTTTATAGACA
	Reverse	TCACATCCTCATACAAACAGCTC
VP6	Forward	GGCTTTAAAACGAAGTCTTC
	Reverse	GGTCACATCCTCTCACTA
VP7	Forward	GTTTAAAAGAGAGAATTTCCG
	Reverse	GGTCACATCATACAATTCTAA
NSP1	Forward	ATGGAAACCATCACCTCCAA
	Reverse	TCTTGTGGTGGCAAATACGA
NSP2	Forward	ATGGCTGAGCTAGCTTG
	Reverse	CCATYTTYTTATCAGTTGAC
NSP3	Forward	ATGCTCAAGATGGAGTCT
	Reverse	GGTCACATAACGCCCCTAT
NSP4	Forward	GGCTTTTAAAAGTTCTGTTCCGAG
	Reverse	GGTCACACTAAGACCATTCC
NSP5	Forward	TTGACGTGACGAGTCTTCCTT
	Reverse	CTTGGTCACAAAACGGGAGT

**Table 2:** Identity of the 11 gene segments of N155 to the corresponding gene segments of strain I321 and other human and bovine rotavirus strains.

Gene segment	I321		Human strains^a^		Bovine strains		Origin of segment in N155
	% similarity	Accession no.	% similarity	Accession no. (strain)	% similarity	Accession no. (strain)	
VP1	NA		86	DQ490551 (RV176-00)	93	DQ870493 (NCDV)	Bovine
VP2	NA		85	DQ870486 (S2)	94	DQ870494 (NCVD)	Bovine
VP3	NA		84	AY277914 (DS1)	92	AY300923 (UKtc)	Bovine
VP4	95	L07657	NA-P[11]		92	M92986 (B223)	Bovine
VP6	97	X94618	87	DQ870507 (DS-1)	94	AB040055 (22R)	Bovine
VP7	93	L07658	88	X63156 (A64)	90	D01056 (KK3)	Bovine
NSP1	97	U08418	89	X59297 (IGV-80-3)	84	DQ494395 (KJ75)	Human
			89	L18943 (Wa)			
NSP2	81	Z47975	95	DQ146656 (Dhaka25-02)	89	DQ494402 (KJ75)	Human
			92	L04534 (Wa)			
NSP3	95	X81433	96	X81436 (ST3)	88	DQ494404 (KJ75)	Human
			94	X81434 (Wa)			
NSP4	95	AY527229	89	AB211989 (RMC/R139)	95	AF166353 (CBNU-1)	Bovine
			83	K02032 (Wa)			
NSP5	NA		92	AB008658 (M318)	95	M33606 (VMRI)	Bovine
			86	V01191 (Wa)			

NA: sequence data not available in GenBank, NA-P[11]: P[11] VP4 sequences detected in humans are likely to be zoonotic transmissions.

a Sequences from strains isolated from humans but for which there is reported evidence of zoonotic transmission and/or reassortment were excluded for homology comparisons.
