# Supplementary material for: Whole genome characterization of reassortant G10P[11] strain (N155) from a neonate with symptomatic rotavirus infection: Identification of genes of human and animal rotavirus origin
Source: J Clin Virol. 2009 Jul;45(3):237–44. doi: 10.1016/j.jcv.2009.05.003 (PMC2913240; doi:10.1016/j.jcv.2009.05.003)
Supplement: Supplementary file 1 [file mmc1.pdf]

Supplementary Table

Complete list and sequences of all primers used for the amplification of the 11 segments

Primers	Primer Sequences	nt Positions	Amplicon Size
**VP1 GENE**			
VP1 BEG F	CTCACAATCCGCAGTTCAAA	70 - 494	424
VP1 BEG R	TTTTCAGCAACGTCATTTTCA		
VP1 P1	GCAGGCTTGACAGTGAATGA	316 -1044	728
VP1 P2	GGCATCCAACATTTTCTGCT		
VP1 G1	CGAGCAATAGTTCCTGACCA	847 - 1311	464
VP1 P4	TCCATTAGCCATGTCATCCA		
VP1 G3	AATGTCATCAGCGTCGAATG	1206 - 2003	797
VP1 P6	ATTCGCGCATACGTTTCTCT		
VP1 G5	GCAGCGAATTCAATAGCAAA	1810 - 2197	387
VP1 P8	TTTCGAATCCTCGAAGCCTA		
VP1 G7	TACACAGTGGGACCAAGCAG	2130 -2803	673
VP1 P10	CTGATTTCGAGCCGTCATCT		
VP1 P11	TCGAGTCAGAAGTCGGGAAT	2392 - 3268	876
VP1 P12	TCGCATTGGTATACGGTGAA		
**VP2 GENE**			
VP2 BEG 1	AAGGTTCAATGGCGTACAGG	9 - 860	851
VP2 BEG 2	CGTCATTCCTTATCCTTTCAGG		
VP2 P1	AGATGCCGGTAAAGTTGTCG	598 - 1444	846
VP2 P2	AACAAAATGCCAGCCAATTC		
VP2 G1	TCATTAATTTCAGGCATGTGG	1223 - 1888	665

VP2 P4	GGCCACTGCATCATTAATCC		
VP2 G3	TGGCATGTGTTACGATGAATA	1692 - 2596	904
VP2 P6	CGATACGAATGCAAGCAGAT		
**VP3 GENE**			
VP3 P1	GGCTCAGGTATATGCGGACA	80 - 977	897
VP3 G2	ATGGGTCCCACGTCTCAAAT		
VP3 P3	GCAATTTGACATCGGTCAGT	805 - 1680	875
VP3 G4	CCAGTTGGTTTTGAGGCTAA		
VP3 P5	ACGCGCTGTCTAACGATTTC	1435 - 2248	813
VP3 G6	ATCCGGCACCATAGAATCTG		
VP3 P7	TGCCGATGATCCGAACTATT	2129 - 2590	461
VP3 P8	TCACGATGTGACCAGTGTGTT		
**VP4 GENE**			
CON3	TGGCTTCGCCATTTTATAGACA	11 - 878	867
CON2	ATTTCGGACCATTTATAACC		
VP4 P1	AATTGCGAGACCGAATGAAG	745 - 1466	721
VP4 P2	GAATTTGCAATTGGAGTCTGG		
VP4 G3	GTGAGCGAACCTCCGTTTAG	1300 - 2100	800
VP4 P4	CACTTCTTCGAATGTTTCTGTCA		
VP4 P5	TGAACAAATTGAATGCTGTGG	1931 - 2351	420
VP4 P6	TCACATCCTCATACAAACAGCTC		
**VP6 GENE**			
VP6 F	GGCTTTAAAACGAAGTCTTC	1 - 1356	1355
VP6 R	GGTCACATCCTCTCACTA		
**VP7 GENE**			

VP7 F	GTTTAAAAGAGAGAATTTCCG	4 - 1013	1009
VP7 R	GGTCACATCATACAATTCTAA		
**NSP1 GENE**			
NSP1 P1	ATGGAAACCATCACCTCCAA	118 - 1002	884
NSP1 P2	CACCATTTGCAATTGTGTACC		
NSP1 P3	TTGAAAACGTCACTCGTTGAA	848 - 1512	664
NSP1 P4	TCTTGTGGTGGCAAATACGA		
**NSP2 GENE**			
NSP2 F	ATGGCTGAGCTAGCTTG	47 - 974	927
NSP2 R	CCATYTTYTTATCAGTTGAC		
**NSP3 GENE**			
NSP3 F	ATGCTCAAGATGGAGTCT	26 - 1050	1024
NSP3 R	GGTCACATAACGCCCCTAT		
**NSP4 GENE**			
NSP4 F	GGCTTTTAAAAGTTCTGTTCCGAG	1 - 750	749
NSP4 R	GGTCACACTAAGACCATTCC		
**NSP5 GENE**			
NSP5 F	TTGACGTGACGAGTCTTCCTT	35 - 667	632
NSP5 R	CTTGGTCACAAAACGGGAGT		
